# Sleepless in Town – Drivers of the Temporal Shift in Dawn Song in Urban European Blackbirds

**DOI:** 10.1371/journal.pone.0071476

**Published:** 2013-08-07

**Authors:** Anja Nordt, Reinhard Klenke

**Affiliations:** Helmholtz Centre for Environmental Research – UFZ, Department of Conservation Biology, Leipzig, Germany; University of Milan, Italy

## Abstract

Organisms living in urban environments are exposed to different environmental conditions compared to their rural conspecifics. Especially anthropogenic noise and artificial night light are closely linked to urbanization and pose new challenges to urban species. Songbirds are particularly affected by these factors, because they rely on the spread of acoustic information and adjust their behaviour to the rhythm of night and day, e.g. time their dawn song according to changing light intensities. Our aim was to clarify the specific contributions of artificial night light and traffic noise on the timing of dawn song of urban European Blackbirds (*Turdus merula*). We investigated the onset of blackbird dawn song along a steep urban gradient ranging from an urban forest to the city centre of Leipzig, Germany. This gradient of anthropogenic noise and artificial night light was reflected in the timing of dawn song. In the city centre, blackbirds started their dawn song up to 5 hours earlier compared to those in semi-natural habitats. We found traffic noise to be the driving factor of the shift of dawn song into true night, although it was not completely separable from the effects of ambient night light. We additionally included meteorological conditions into the analysis and found an effect on the song onset. Cloudy and cold weather delayed the onset, but cloud cover was assumed to reflect night light emissions, thus, amplified sky luminance and increased the effect of artificial night light. Beside these temporal effects, we also found differences in the spatial autocorrelation of dawn song onset showing a much higher variability in noisy city areas than in rural parks and forests. These findings indicate that urban hazards such as ambient noise and light pollution show a manifold interference with naturally evolved cycles and have significant effects on the activity patterns of urban blackbirds.

## Introduction

Mankind has altered Earth towards its requirements to an extent which caused Crutzen to rename the last three centuries the ‘Anthropocene’ – the human-dominated, geological epoch [1], [2]. The conversion of natural landscapes to built-up area is one of the tremendous changes humans caused in the last three centuries [3]. At the end of the 20^th^ century less than 3% of the Earth’s terrestrial surface was covered by human settlements, but the impacts were rather global as they consume 60% of residential water, 76% of industrial used wood, and contribute 78% to carbon emissions [4], [5]. In the foreseeable future, humans will become increasingly urban [6] and cities will further increase in both extent and inhabitants as population growth is expected to concentrate in urban settlements [7].

With regard to flora and fauna, cities constitute a novel environment where organisms are exposed to other ecological conditions than their rural conspecifics [8], [9]. These are, amongst others, higher temperatures than in the surrounding, chemical contaminants, modified habitat structure including sealed pavement, concrete buildings, and exotic vegetation [9]. Moreover, anthropogenic noise and artificial night lighting are two major challenges closely linked to urbanization.

Although ambient noise is ubiquitous in many habitats [9], [10] in terms of leaf-rustling, waterfalls or animal sounds, anthropogenic noise is novel by its high amplitude, low frequency, and diurnal periodicity [11]. Anthropogenic noise interferes with the spread of acoustic information and, thus, is associated with a variety of fitness and behavioural consequences. Recently, Schroeder et al. [12] demonstrated that House Sparrows (*Passer domesticus*) breeding under noisy conditions experience significant fitness consequences due to impaired parent-offspring communication. Further studies suggest that several oscine species change temporal and acoustic traits of their song to avoid masking by anthropogenic noise: Nightingales (*Luscinia megarhynchos*) [13], Song Sparrows (*Melospiza melodia*) [14], Great Tits (*Parus major*) [15], [16], and European Blackbirds [17], [18], [19], whereas European Robins (*Erithacus rubecula*), Chaffinches (*Fringella coelebs*), Blue Tits (*Cyanistes caeruleus*), and also Great Tits advance their song onset into true night with quieter conditions [11], [20]. If they cannot modify their song characteristics, bird species with low-frequency vocalization should have difficulties to communicate in the presence of anthropogenic noise. A possible strategy might be to abandon these noisy but otherwise suitable habitats [21], which would lead to a changed composition of the bird community in noisy areas [e. g. 9,22,23]. However, few studies investigated the effect of noise on behaviour and fitness considering possibly confounding factors, like artificial light, which are usually associated with anthropogenic noise.

Artificial night light is such a factor that has long been neglected [24] and reached awareness in science and policy only recently [25], [26]. Most species evolved under the daily cycle of day and night and developed molecular mechanisms to synchronize their internal clock to the photoperiod [27] to optimize timing of foraging, communication, reproduction, and migration [28]. Therefore, light pollution, the alteration of naturally dark sites by artificial light, disrupts animal behaviour, physiology, and ecological interactions across a wide range of taxa [24]. For instance, artificial light attracts moths over long distances [29]. These prey aggregations at street lights are readily exploited by fast flying bats, whereas slow-flying bats avoid lights due to increased predation risk [30] which might result in a shift at the community level. Intensively illuminated structures also cause migrating birds to become disoriented and entrapped in the light cone, thus disturbing migratory behaviour [31], [32], [33], [34]. Furthermore, light pollution is suspected to interfere with avian circannual and circadian rhythmicity, because photoperiod is one of the most important cues in timing seasonal and daily activities [35], [36]. European Blackbirds advance the growth of their testes by one month after experimental exposure to low artificial night light [37]. In artificially lit territories, female Blue Tits start egg laying 1.5 days earlier than females in unlit territories [38], whereas Great Tits increased provisioning of their nestlings after a light was installed on the nest box which was interpreted as a misperception of a prolonged photoperiod [39]. In many songbirds, male singing activity peaks before sunrise to attract mates and defend the territory [40]. This dawn song is closely linked to changing light intensities [41]. Hence, night singing of diurnal birds close to artificial light sources is often regarded as a consequence of light pollution [38], [42], [43].

With few exceptions [e. g. 11], most studies of night singing in birds investigate only one anthropogenic factor and, consequently, shifts in the timing of dawn song are attributed either to anthropogenic noise or to artificial night light. However, especially in urban settings, traffic noise is the largest single contributor to anthropogenic noise [44] and most streets are lined with street lamps for safety reasons. Thus, the anthropogenic factors noise and night light are not completely separable from each other. To analyse the impact of only one or the other cannot contribute to a comprehensive understanding of the underlying interdependencies. Here, we investigate in a combined approach the specific contributions of artificial night light, traffic noise and weather conditions on the timing of dawn song of urban dwelling European Blackbirds.

Originally, the blackbird inhabited dense forests, but expanded into European cities since the early 19^th^ century [45], where it is today one of the most common species [46]. Urban blackbirds exhibit some traits which were seen as adaptations to city life, although it is still under discussion whether these traits are based on genetic differentiation between urban and rural populations or whether they are within the range of behavioural plasticity [47], [48]. Urban birds have higher breeding densities, an extended reproductive period, are more stress tolerant and tamer than their rural conspecifics, and show reduced migratory behaviour because of continuous food availability and higher temperatures in the city during winter [8], [42], [48], [49], [50]. We have chosen this medium sized thrush as a model organism, because the males exhibit a strong morning chorus, which is extended into the night hours by urbanites [48], whereas rural birds do not sing during true night [42].

## Methods

### Study Area

**Figure 1:**
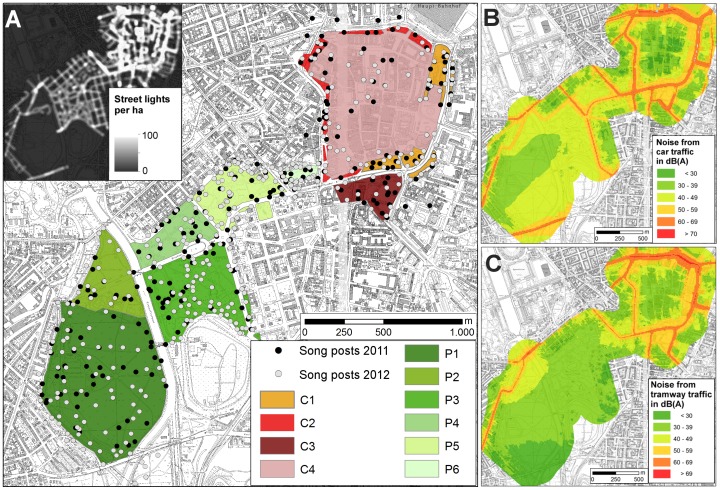
Study area in Leipzig with artificial night light (A), noise from car traffic (B) and tramways (C). (**A**) represents the study area with its different sites (C1–P6). In the northeast of the study area are the inner city centre (C4) with adjacent tiny parks (C1) and green spaces (C2) and an urban fallow area (C3). To the southwest follows a series of public parks of different sizes (P2– P6) and the urban forest (P1). The whole study area covers 215 ha. The black and white points indicate blackbird song posts in the two seasons 2011 and 2012, respectively. One bird can use more than one song post. The inlay in (A) illustrates the street light densities of the study area and its close vicinity. Noise levels for car traffic (B) and tramways (C) at night are shown for the same area. Noise levels above 60 dB(A) are indicated by orange and red colour.

The study took place in the city of Leipzig, Germany (51°20′ N, 12°25′ E), which is unique for the riparian forest crossing the centre on north-south direction. Thus, natural and semi-natural habitats occur in close vicinity to non-natural habitats of the city. Out of the semi-natural habitats (urban forest, parks, P) and non-natural habitats of the city (C), we selected ten study sites spanning a gradient of noise and light intensity (Fig. 1 & Fig. S1). The inner city centre itself is a traffic-calmed pedestrian zone and thus exceptionally quiet, so it deviates from the pattern of an increasing noise and light level from semi-natural to non-natural habitats (C4, Fig. S1). The inner city centre is well defined by a noisy and busy ring road with adjacent tiny parks (C1) and green spaces (C2). A derelict area used as parking lot marks the southern edge of the city (C3). To the southwest follow a series of public parks (P2– P6) of different sizes with pathways and lawns and a great variety of evergreen and deciduous bushes and trees. A dense urban forest (P1) links those parks to the riparian forest. The successive transition from semi-natural to urban structures stretches over only 3 km representing a steep urban gradient. The whole study site covers 215 ha and is surrounded by residential housing.

### Study Period

We collected data of the onset of blackbird dawn song for ten weeks in 2011, from 19 March to 24 May, and for five weeks in 2012 from 12 March to 18 April. In 2011, the study period was divided into a first period of intensive sampling over 14 consecutive nights (19 March –01 April 2011) and a second period with observations once per week (05 April –24 May 2011). We choose the first period to cover a change in human activity, as winter time was changed to daylight saving time on 27 March 2011. After this change, human activity and, hence, the rise of noise levels started one hour earlier with regard to sunrise. To test the effect of this earlier rise in anthropogenic noise levels we compared the song onset times of the first week (winter time) of observations in 2011 with those in the second week (daylight saving time). This analysis was restricted to the weekdays from Monday till Friday since human activity is not as concentrated in rush hours on weekends as during the work week.

To test for changing influences in the course of the breeding period and with shorter nights, each study site was examined once per week in the second period of 2011, and three nights per week in 2012. This accounted to additional eight, respectively fifteen survey nights. In total, every study site was investigated for 37 survey nights.

### Blackbird Song Measurements

The mapping of song posts took place in the two weeks prior to the main study in both seasons. Individual song posts of blackbirds were mapped during the morning peak of singing activity between 2 a.m. and sunrise. A song post was identified by localizing the singing blackbird either by sight or hearing. Song posts were stated as one single post when they were closer than 10 m to each other (e. g. different branches of the same tree). Every study site was visited at least four times during the preliminary study to ensure that all established song posts are identified. Subsequently, territory owners could be distinguished from each other by special song characteristics, territory location and their preferred song posts. In total, 237 (2011) and 363 (2012) song posts of 127 and 314 blackbird territories were included into the study with, on average, 1.4±0.6 song posts per territory. Table 1 and Figure 1 give an overview of the number and distribution of song posts at the different study sites.

**Table 1:** Number of blackbird song posts at the different study sites.

		Centre				Park & Forest					
Study site		C1	C2	C3	C4	P1	P2	P3	P4	P5	P6
**Season**	2011	22	46	16	10	52	8	37	17	21	8
	2012	30	20	14	26	83	18	69	23	47	33

Each survey of blackbird song onset started at 1∶30 a.m. and lasted until sunrise. During a survey night, each blackbird territory was visited repeatedly every 24.4±20.6 min, on average, until song activity was observed. A territory owner was stated to be active when it sang from one of its song posts. When an observer was within hearing range of a territory and noticed that a bird started to sing after more than five minutes of silence, we defined this as the song onset of this individual blackbird. This applied to 491 out of 3061 observations. For the remaining observations, it was not possible to determine the actual song onset more precisely than the period between the last visit to the territory with registered inactivity and the proximate visit with observed song activity. We assumed that the probability of a bird to start singing was equal for every time point between these consecutive visits. Therefore a random time in this period was chosen and further referred to as song onset. The so defined song onset times were stated as minutes before sunrise to standardize the observations and to avoid a bias caused by increasing day length.

To avoid a bias of approaching the territories always in the same order we changed the route between the territories each surveyed night.

### Environmental Parameters

Different sources of noise contribute to the overall noise load. As the study area is only affected by noise from car traffic and tramway traffic, we restricted the analyses to noise from these two sources. Values for car and tramway noise at the individual song posts were obtained from night-time noise indices which were based on traffic counts and specified in dB(A). These night-time noise indices were calculated for the time between 10 pm and 6 am separately for each source of noise, indicate noise levels at 4 m height, include first order reflections on e. g. walls, and incorporate extensive correction factors for e. g. speed limit, slope, paving, and noise absorbing or reflecting facades of buildings [51]. A detailed description of the computation specifications including formulas are given in [51]. These indices were conducted to comply with reporting commitments under the EU directive of ambient noise [52] and were kindly provided by the Environmental Protection Office Leipzig. These noise indices provide absolute values of night-time noise which are independent of the steady increase towards the morning rush hour. During the study period, blackbird dawn song started considerably before the morning rush hour, therefore we considered these indices of night-time noise as an appropriate measure to study the effects of night-time noise on the onset of blackbird dawn song.

Artificial night light is composed of many sources of light (streetlights, lights emitted from windows, illuminated advertisement, cars, etc.). We used the density of municipal street lighting as a proxy for artificial light intensities because street lighting is the largest single contributor to urban artificial night light [53] and is relatively stable throughout the night. Streetlights in the study area are mainly 70 watt high-pressure sodium lamps (pers. comm. M. Mahler; pers. obs.), which emit a yellow-orange light. Light point data were kindly provided by the municipal traffic and works service, Leipzig. Based upon these data we calculated a lamp density map using kernel density estimation with a search radius of 50 m and a resolution of 10 m (ArcGIS 10, ESRI). Values of the lamp density were extracted for each individual song post and used for further analyses. We verified the accuracy of this estimation of ambient night light by directly measuring the illumination at 100 randomly chosen points in the study area with a lightmeter (LM 37, TFA Dostmann, resolution 0.01 lx). The measurement was conducted during a moonless and starlit night to avoid interference with moonlight or reflections from clouds. At every point location the illumination was measured with the external sensor pointing upwards. Measured data and the calculated street lamp density of the same 100 random points correlated significantly (Pearson’s r = 0.629, df = 98, p<0.001). Thus, we regard the density of streetlights as an accurate indicator of artificial night light.

The German Weather Service (Deutscher Wetterdienst, DWD) provided data for temperature (in °C) and hourly measurements of cloud cover (in oktas). Local lunar phases (percentage of disc visible), daily civil twilight and sunrise times were calculated from formulas given in Duffet-Smith and Zwart [54].

### Data Analysis

In a first approach we applied a Repeated Measures ANOVA to test whether the blackbirds differed in their song onset times between the study sites. S*tudy site* was included as a factor. To account for the dependency of repeatedly observing the same birds, *territory* was included as a repeated measure.

We used a general linear mixed model (LMM) to investigate which variables affect the timing of blackbird dawn song. To account for the effect of changing lengths of day over the course of the observation period the Julian date was incorporated. Tramway noise correlated considerably with traffic noise and, thus, violated the presumption of independency between explanatory variables. To include both sources of noise we conducted a Principal Component Analysis (PCA) of the variables traffic noise and tramway noise. The resulting PCA factor had an eigenvalue of 1.6 and explained 81.9% of the total variance in the noise data. The factor loadings were 0.91 both for traffic and tramway noise. Using the PCA factor reduced the amount of explanatory variables. In the following, it is referred to as the explanatory variable noise. After this conversion, a variance inflation factor (VIF) below 2 for all explanatory variables revealed independency of all remaining variables [55]. To remove the influence of different levels of variance between the variables the further continuous explanatory variables *light density*, *Julian date*, *cloud cover*, *temperature*, and *lunar phase* were standardized by subtracting the mean and dividing by its standard deviation.

The LMM included the habitat (city (C1–C4) vs. semi-natural habitats (P1–P6)), *PCA factor noise*, *lamp density*, *cloud cover*, *temperature*, *lunar phase*, and the *Julian date* as explanatory variables. Furthermore, the full model also contained the interactions between *noise* and *lamp density* as well as between *cloud cover* and *lamp density*. A factor *territory* was included as a random intercept to account for the non-independence of repeated measurements of the same birds. A spatial residual correlation structure was added to the LMM [55] to account for the stimulating effect of an already singing bird on nearby conspecifics. An analysis of spatial autocorrelation was conducted additionally. We used correlograms and Moran’s I to get more insight into the spatial processes.

We based model selection on Akaike’s Information Criterion (AIC) to choose a model from the candidate set [56]. ΔAIC values were calculated as the difference between the AIC of each model and the AIC of the best model. We considered candidate models with ΔAIC <4 to receive competitive support and included them to calculate Akaike weights (*ω_i_*).

As the differentiation between the semi-natural habitats (P1–P6) and the non-natural habitats of the city (C1–C4) revealed to have an influence, the analyses were rerun for each spatial subset separately.

We applied a Repeated Measures Analysis of Covariance (Repeated Measures ANCOVA) to test for an effect of the advanced rise in noise levels after the change to daylight saving time on the onset of blackbird dawn song. To differentiate between song onset times prior and after the clock change a factor *change* was included into the analysis. The variable *weekday* was defined as covariate. The factor *territory* was, again, included to account for repeated measurements of the same birds on different weekdays.

We performed all statistical analyses with the R software system version 2.15.2 [57]. LMMs were computed with the R-libraries *mgcv* [58] and *nlme* [59]. For spatial statistics we used *sp* [60], [61], *spdep* [62], *gstat* [63], *ade4* [64], and *maptools* [65].

### Ethics Statement

Ethics approval was not required for any of the research described in this study because no bird was handled or otherwise influenced by the study design. The study was conducted in public area exclusively, where no permit was required for accessing the sites.

### Data Archiving

Data used in this paper will be archived at the Dryad data repository (http://dx.doi.org).

## Results

### Noise and Night Light

Singing blackbirds were heard at 597 different song posts. On average, the birds were exposed to the light from of 8.48±8.3 (Mean ± SD) street lamps in a 50 m radius and experienced 47.6±8.0 dB traffic noise and 41.1±10.5 dB tramway noise at the song posts. These site characteristics differed significantly between the study sites (ANOVA, *traffic noise*: F_9, 598_ = 59.8, p<0.001; *tram noise*: F_9, 598_ = 174.0, p<0.001; *lamp density*: F_9, 598_ = 56.0, p<0.001, Fig. 1 & Fig. S1). Toward northeastern direction, the noise and light levels steadily increased, representing a steep urban gradient. Only site C4, the inner city centre, deviated from this pattern because it is traffic calmed and thus exceptionally quiet.

### Onset of Dawn Song

**Figure 2:**
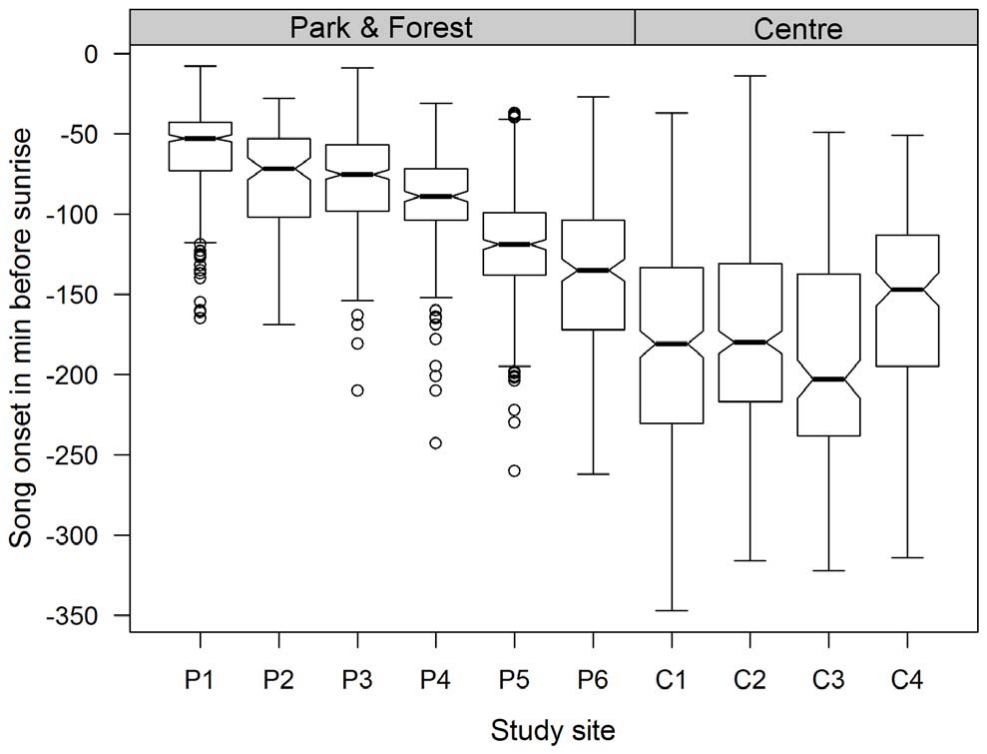
Onset of blackbird dawn song along an urbanisation gradient. If the notches of two boxes do not overlap it is strong evidence that their medians differ.

In areas of no artificial night light or traffic noise blackbirds were never heard to sing during true night. However, the brighter and noisier a site was during the night, the earlier the blackbirds started to sing. Blackbirds in the city centre started their dawn songs significantly earlier than their conspecifics in the parks and forest (Repeated Measures ANOVA, F_9, 2611_ = 192.3, p<0,001; Fig. 2). City birds sang up to 5∶31 hours earlier than the latest in the urban forest (P1). On average, the time difference between the first and the last bird in a survey night, accounted to 3∶43±0∶48 h. The earliest singing males were situated next to the ring road (C1–C3). Blackbirds in C4 started significantly later than their nearby conspecifics in C1– C3 despite these sites have similar night light intensities. However, compared to the song onset of forest blackbirds (P1) the city centre birds start far earlier although they experience similar levels of traffic noise (Fig. S1). Birds at all four city centre sites were very variable in their song onset times. Contrarily, the birds in the parks and forest (P1– P6) showed a more stable onset and a distinct temporal succession in the song onset times (Fig. 2). With a mean song onset of 59.8±25.5 min before sunrise, forest blackbirds (P1) initiated their dawn song close to the break of civil twilight. At this time, the sun is still 6° below the horizon, but sunlight scattered from the atmosphere is sufficient to discriminate contours and the horizon becomes visible. Morning civil twilight ends with the sunrise.

**Figure 3:**
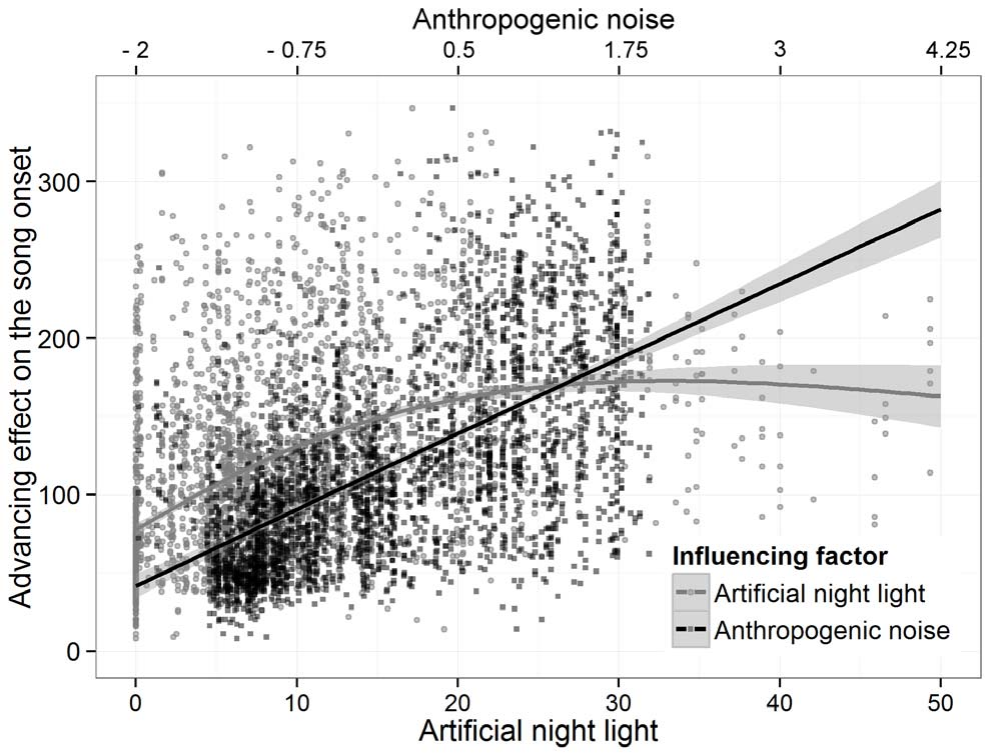
Proposed influence of anthropogenic noise and artificial night light on the song onset of blackbirds. The smoothing function of anthropogenic noise (here derived from the PCA-factor noise) is a linearly increasing effect on the song onset of blackbirds, whereas the smoothing function of artificial night light (street lamps per ha) approximates an asymptote. The smoothing functions are plotted with the 95% Confidence Interval.

**Figure 4:**
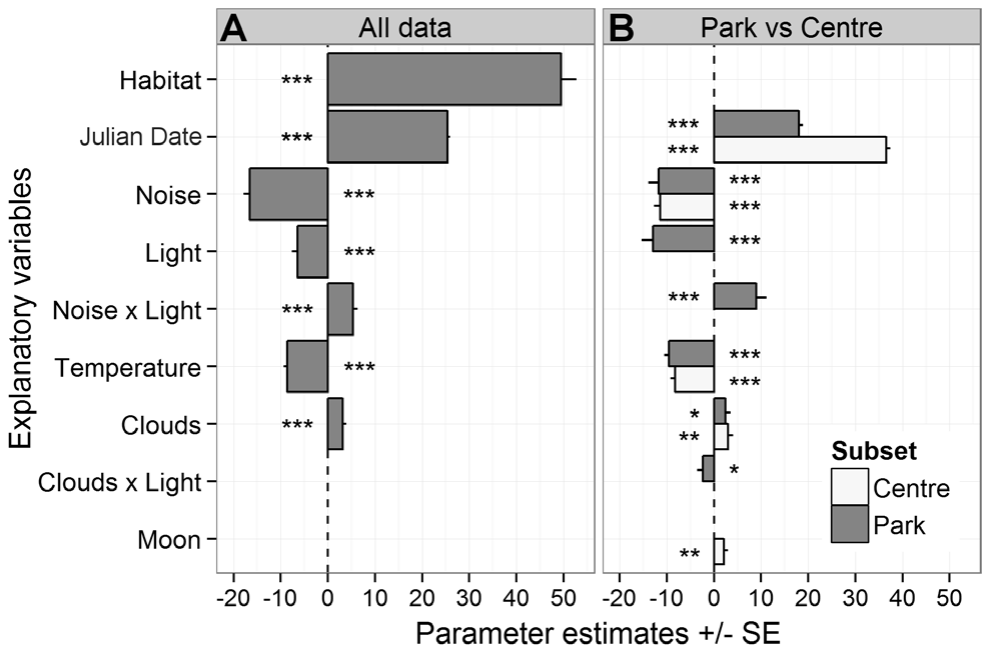
Parameter estimates for the influence of explanatory variables on the onset of blackbird dawn song. The panels illustrate the best models of the (A) overall data set and (B) park/forest and city centre subsets. Negative values indicate an advancing effect on the song onset, positive values a delaying effect. Only explanatory variables included in the best models are shown here. Asterisks represent the levels of significance for the explanatory variables as follows ***P<0.001, **P<0.01, *P<0.05.

The variance in song onset times was best described by a candidate model including the main effects of *Julian date*, *habitat type*, *noise*, *lamp density*, *cloud cover* and *temperature*, and the interaction of *noise* with street *lamp density* (Table 2). The factors of anthropogenic origin, noise and street lamp density, had a significant advancing effect on the onset of dawn song (Fig. 3 & 4a). The more noise or artificial night light occurred in the surrounding of a song post, the earlier the blackbirds initiated their dawn chorus. Anthropogenic noise, though, was twice as effective as artificial night light. These effects were further amplified by interacting traffic noise and night light. Low temperature and an overcast sky delayed the initiation of dawn song significantly.

**Table 2:** Model selection results for the subsets and full dataset examining the influence of anthropogenic noise, artificial night light and weather components on the onset of blackbird dawn song.

Subset	Candidate models^a^	*df*	*K*	AIC	ΔAIC	*ω_i_*
All data						
	Noi, Ld, Day, Temp, Cloud, Habitat, Noi*Ld	2613	11	29917.7	0.00	0.43
	Noi, Ld, Day, Temp, Cloud, Habitat, Noi*Ld, Cloud*Ld	2612	12	29918.6	1.03	0.26
	Noi, Ld, Day, Temp, Cloud, Habitat, Moon, Noi*Ld,	2612	12	29919.3	1.61	0.19
	Noi, Ld, Day, Temp, Cloud, Habitat, Moon, Noi*Ld, Cloud*Ld	2611	13	29920.3	2.62	0.12
Centre						
	Noi, Day, Temp, Cloud, Moon	891	9	9751.7	0.00	0.40
	Noi, Ld, Day, Temp, Cloud, Moon	890	10	9751.7	0.04	0.39
	Noi, Ld, Day, Temp, Cloud, Moon, Cloud*Ld	889	11	9753.6	1.91	0.15
	Noi, Ld, Day, Temp, Cloud, Moon, Noi*Ld, Cloud*Ld	888	12	9755.6	3.90	0.06
Park						
	Noi, Ld, Day, Temp, Cloud, Noi*Ld, Cloud*Ld	1714	11	19798.8	0.00	0.47
	Noi, Ld, Day, Temp, Cloud, Moon, Noi*Ld, Cloud*Ld	1713	12	19799.1	0.34	0.40
	Noi, Ld, Day, Temp, Cloud, Noi*Ld	1715	10	19801.3	2.49	0.13

All models contain a correction factor for spatial autocorrelation and a random intercept to account for repeated measures of individual birds.

a Noi – PCA factor noise, Ld – lamp density, Day – Julian date, Temp – temperature, Cloud – cloud cover, Moon – lunar phase; Habitat – habitat factor.

Model selection was based on Akaike’s information criteria (AIC). The parameter *df* indicates the residual degrees of freedom, *K* represents the number of parameters in the candidate model and ΔAIC the difference of AIC values to the top-ranking model. Only candidate models with ΔAIC <4 are presented, because they are considered to receive support, and included to calculate Akaike weights (*ω_i_*).

The strong influence of the factor habitat, which separated the centre from the park and forest, indicated possibly different processes in the two subsets. When we restricted the analyses to either the city centre or the park and forest subset, we received slightly different results. Whereas weather and noise were still determining factors of song onset for blackbirds in the city centre, the lamp density and its interaction with noise were not (Fig. 4b). Song initiation times instead co-varied with the lunar phase.

On the other hand, timing of blackbird dawn song in the parks and forest was again correlated to *noise*, *lamp density*, *temperature* and *cloud cover* (Fig. 4b). On overcast mornings, park blackbirds delayed their song onset by approximately a quarter of an hour. However, in artificially lit park areas, the birds started significantly earlier to sing under cloudy conditions as indicated by the significant interaction between *clouds* and *lamp density*.

In the course of the study period, true night shortened by approximately 4 min per day. Although we corrected the song onset times for the influence of the shortened nights by calculating the time differences until sunrise, we found a significant pattern related to the progress in season which was indicated by the explanatory variable *Julian date*. This pattern suggests that birds did not keep up with this time difference but started their dawn song less early and thus significantly closer to sunrise with the progress of the season (Fig. S2). This pattern was significant in the best models of both spatial subsets, but more pronounced in the city centre. The inactive resting time decreased to 6 hours per night towards the end of the study period.

### Spatial Autocorrelation

A detailed analysis of the spatial correlation in different distances revealed that the spatial patterns and probably also processes are completely different between the spatial subsets of data from parks and forest on the one hand and the city centre on the other hand. The global Moran’s I is 0.549 (statistic standard deviate = 18.45, p<0.001) for the sub-sample of parks and forest and 0.092 (statistic standard deviate = 2.57, p = 0.005) for the city centre.

Correlograms of the song onset for the two spatial subsets using Moran's I for different distance classes (Fig. S3) indicate that blackbirds in parks and forest start to sing rather homogeneous up to a distance of approximately 500 m, whereas males in the city centre showed only a very low spatial autocorrelation in a distance below 50 m. Although we find some significant clustering in the parks and forest the correlation between starting times in distances above 100 meters is also rather weak. Interestingly, the results further indicate that city blackbirds which are separated by 1000 m show similar song onset times. The city centre has a diameter of approximately 1000 m and, thus, the blackbirds at the ring road at one side of the centre fall into this category when they are compared with those blackbirds at the other side of the centre (Fig. 1). Similarly, the correlated distance category of 2500 m for park blackbirds links P6 to the southernmost point of the forest (P1), which adjoins an illuminated and busy road (Fig. 1 A & B).

### Time Shift

After the change to daylight saving time, noise levels were assumed to rise one hour earlier due to the advanced human activity. This affected the onset of blackbirds only marginally. A tendency to alter the onset of dawn song after the change to daylight savings time was found only for birds at the study site C2 (Repeated Measures ANCOVA, *C2*: *change*: F_1, 87_ = 5.53, p = 0.029, *weekday*: F_1, 87_ = 6.17, p = 0.015, change*weekday: F_1, 87_ = 5.19, p = 0.025 ). The difference accounted to −17.7±7.5 min for the Intercept and 5.02±2.20 min per day in the slope, respectively. These values indicate that on Monday after the change blackbirds started 12.6 min later than during the previous week. This would be in line with the observed delay from day to day. Nevertheless, they gradually advanced their song onset until Friday when they started 7.4 min earlier than prior to the clock change.

## Discussion

### Noise Annoys but Light Kills the Night

Our study demonstrated that the heterogeneity of street lamp density as an indicator of artificial night light, and noise conditions were reflected in the timing of blackbird dawn song. Under natural conditions, the blackbird dawn chorus coincides with the break of civil twilight [66]. Changing light intensities serve as the primary zeitgeber and trigger the endogenous circadian rhythm [41], [67]. Beyond this, the time of song onset is not fixed but subject to further environmental variations. We found that cloud cover delayed song onset presumably because it reduced the sky radiance and, thus, the critical light intensity was reached later. Furthermore, another meteorological component, temperature, markedly affected song initiation. Low temperatures delayed dawn song significantly. These results confirm those from earlier studies [e.g. 42,67,68].

Anthropogenic noise and artificial night light interfere with these processes and prompt birds to sing earlier. Urban blackbirds were thought to have become adapted to the high noise levels in the city [42]. Accordingly, our results indicate that anthropogenic noise contributed considerably to the temporal shift in dawn song: the higher the noise level, the earlier the blackbirds started to sing. This can be seen as a possible adaptation to the city noise in terms of temporal avoidance. There are two explanations for the birds’ responses to the different noise levels: either (i) noise acts as a wake-up stimulus [8], [69] or (ii) the birds avoid direct interference and hence shift their song activity to time slots of quieter conditions before noise can act as a wake-up stimulus, namely the night and early morning hours [11]. After the change to daylight saving time, only blackbirds at the noisy ring road (C2) tended to gradually advance the dawn chorus by some minutes until the end of the week. At all other sites we found no effect. This is certainly not a clear evidence for one or the other hypotheses. If birds respond to noise because they avoid the masking of acoustic information, they should know in advance when noisy or quieter conditions will occur. Under predictable conditions this might be achievable. Furthermore, several oscine bird species, including blackbirds, are capable of vocal adjustments that minimize masking by ambient, low-frequency noise: they increase the pitch of their song, twitter proportionately more, pause longer or just sing louder in urban environments [16], [18], [19], [70], [71], [72]. These adaptations may be a consequence of behavioural plasticity [14], [73], whereby only singing at a higher amplitude might cause some extra costs [74]. Nevertheless, birds might be confronted with even higher energetic costs when singing rather than sleeping during the night [75]. Therefore, we suggest that if signal transmission could be ensured by adjusting acoustic parameters, it would be more beneficial to deal with the noise instead of avoiding it.

Because blackbirds, nevertheless, sang during true night, energy was either not a limiting factor or other constraints than noise need to be considered. In fact, our results indicate that artificial night light advances the song onset, too. Anecdotal reports mention that artificial night light stimulates passerines to sing during the night [42], [76], [77]. Two recent studies focus exclusively on the effects of light pollution on different oscine species [38], [43]. Concordant with our findings, blackbirds and other early-singing species respond by considerably advancing their song onset under artificial night light [38]. As we found in our study, too, this effect is further amplified by cloudy conditions, assumedly because artificial light is reflected by the clouds which made the night even brighter [43], [78], [79]. However, the results of Miller [43] and Kempenaers et al. [38] are somewhat limited because anthropogenic noise was not considered or stated to be absent. Another study included traffic noise into the analysis and found it to be the sole driving factor of night-time singing in European Robins [11]. Although their data would also support that night-time singing occurred only above a threshold of 1 lux of artificial night light, no significant effect of artificial night light was found after controlling for the effect of noise. In this study of night-singing in European Robins [11] exclusively urban sites were compared, which resembles our city centre subset. Similarly, at our sites of highest ambient night light, the city centre, street lamp density and its interactions also failed to explain much variance in the song onset times. Although these sites still showed a high variability in the amount of night light, it might be beyond a threshold, needed to further advance the onset of singing. A recent study provided experimental evidence that artificial night light as low as 0.3 lux has the potential to cause a significant advance in song onset and reproductive physiology [37]. Added to this, artificial night light at a certain site was uniform throughout the night. It was therefore comparable to the situation at high latitudes, where the variability of (natural) light is low and not anymore a reliable cue [80]. Thus, the influence of secondary zeitgebers like acoustic stimuli and temperature increases [20], [67]. For these reasons, a perceivable effect of light pollution seemed to be restricted to low and intermediate artificial light levels, while high levels might lead to an enhanced alertness of the birds, which subsequently resulted in a stronger response toward other environmental impacts, especially anthropogenic noise.

### Seasonal Differences

With the proceeding season, the observed song onset times did not keep up with the declining night length but birds started their dawn song significantly later with regard to sunrise. Stephan [42] already described this pattern. The importance of singing early has been higher at the beginning of the season than later. In general, song activity is most pronounced during territory establishment and female attraction [e.g. 81], or rather around mate fertility [82], [83], [84], [85]. Among other song features, such as repertoire size, song rate and song duration [86], [87], [88], singing early is hypothesized to be a reliable indicator of male quality [38], [88], [89], [90], [91]. Cuthill and MacDonald suggest a potential explanation [82]: the night prevents diurnal birds from foraging and replenishing their energy reserves, only those males which accumulated enough energy resources before can invest time in extensive dawn song instead of initiating foraging as soon as light intensities permit. Females determine male quality indirectly by assessing these song traits. The selective pressure to display one’s quality and, thus, the need to sing early diminishes when territories are established, pair bonds are formed, and eggs are laid. Although breeding state of the focus males’ mates are not included in our study, Cuthill & MacDonald [82] found male blackbirds to advance their song onset until the day their mate laid the first egg. Afterwards they clearly delayed song onset through the laying period. Similarly, under natural light conditions only unpaired Northern Mockingbirds (*Mimus polyglottos*) sang extensively during the night [92].

Alternatively, the delay in song onset over the season is due to minimum requirements of rest during the night. At our latitudes, true night shortened from 10∶50 h to 6∶34 h between start and end of our study. Assuming that a bird in the city centre roosted by the end of civil twilight and started singing at an average time, the resting times per night at the beginning, mid and end of the study would be 7∶38, 6∶49 and 6∶12 h, respectively. Observations during polar day of European Starlings (*Sturnus vulgaris*) revealed that they pause offspring-feeding for approximately 6∶30 h during the night, whereas House Martins (*Delichon urbica*) are inactive for only 4 to 5 hours [93]. Similarly, Great Tits spent proportionally less daytime active in higher latitudes [94]. This indicates that birds need to roost even though light intensities, irrespective if of natural or artificial origin, would permit continued activity [43], [95]. The length of the inactive period is highly species dependent [96]. From our data we suggest that the minimum for blackbirds in Leipzig is approximately 5 to 6 hours per night which would be in line with Szymczak et al. [97]. Whether a reduced necessity to display male’s quality or the need to sleep caused the observed seasonal delay of song onset, cannot be clarified conclusively here, particularly, because the two explanations are not mutually exclusive.

### Spatial Autocorrelation

Social cues act as a minor zeitgeber in a wide array of animals, including birds, to fine-tune timed responses in the context of social interactions [98]. In several bird species, where timing of dawn song advertises male quality, an already singing bird has a stimulating effect on nearby competitors [42], [99]. Therefore, we expected to find little variation in the song onset times between close neighbours. Indeed, this was the case for the park and forest birds but, contrarily, the city centre birds varied significantly in their song onset. At small scale, artificial night light and anthropogenic noise are highly variable in the city. House walls block or enhance noise and artificial light depending on their orientation to the source and the song post. In the parks and forest, few buildings interfered with the propagation of noise and light and conditions were relatively homogenous over extended areas. Thus, our results indicate that the observed patterns of high variability in song onset times in the city and low variation in the parks were rather caused by the different amount of variance in the determining factors noise and night light. The correlation of song onset times of blackbirds whose song posts are 1 km (city) or even 2.5 km (park) apart from each other, further support this hypothesis. Park blackbirds, supposedly, did not awake by the song of a conspecific, but due to noise and light conditions. Social cues prompt them to sing earlier only once they are awake, as already supposed by Foote et al. [99]. In this case, similar environmental conditions caused low variability in song onset times and hence induced a spatial autocorrelation [100].

### Conclusion and Outlook

Our results showed that the anthropogenic factors noise and night light interfered with the natural processes determining the timing of blackbird dawn song. A possible consequence was described by Kempenaers et al. [38]: yearling blue tits with territories close to street lights were chosen disproportionately often by females for extra-pair copulations. Due to the light-induced early singing the yearlings were misperceived as high quality males. This may have far reaching detrimental effects on population dynamics, as well as enhanced energy expenditures or predation risks due to nocturnal singing [43]. We propose a possible correlation between artificial noise, night light and the song onset: noise advanced the song onset in a rather linear way, whereas artificial night light reached an asymptotic value and additional brightness did not contribute to further advancing the song onset times. In a broader context, the influence of low night light may be even more disruptive [37]. Black-tailed Godwits (*Limosa l. limosa*) prefer nest sites with a minimum distance of 300 m to artificially lit roads [101]. Thus, already low levels of artificial night light have the potential to interrupt the natural evolved cycles and are harmful in critical life stages. For example, juvenile Cougars (*Felis concolor*) use corridors for dispersal only when these lack artificial outdoor lighting [102]. This needs to be considered when establishing new conservation areas. Today, many nature reserves are exposed to sky glow from nearby urban areas, which further threatens endangered species [28]. Ongoing urbanisation and the increasing use of LEDs with spectra shifted towards shorter wavelengths which are physiological more active [103], will probably multiply the observed impacts [79], [104].

## Supporting Information

Figure S1 **Study sites differ in traffic noise and artificial night light.** The amount of traffic noise and artificial night light blackbirds experience at the song post are plotted as the mean and standard error for the corresponding study site. In the city centre, the sites show a high variability, whereas the bigger parks (P2– P5) are relatively homogenous. From the urban forest (P1) to the green spaces next to the ring road (C2) the noise and night light steadily increases, only the inner city centre deviates from the pattern by its low traffic noise.
(TIFF)

Figure S2 **Delay of song onset of park and city centre blackbirds over the study period.** A Julian Date of 90 indicates the 1^st^ April 2011 and the 31^st^ March 2012, respectively.
(TIFF)

Figure S3 **Moran’s I computed by distance class.** Positive (negative) values indicate positive (negative) spatial autocorrelation. Values range from −1 (indicating perfect dispersion) to +1 (perfect correlation). A Moran’s I of zero indicates a random spatial pattern. Red points indicate spatial autocorrelation that is significant at the 5% level.
(TIF)
