# Supplementary figures and images for: Sleepless in Town – Drivers of the Temporal Shift in Dawn Song in Urban European Blackbirds
Source: PLoS One. 2013 Aug 7;8(8):e71476. doi: 10.1371/journal.pone.0071476 (PMC3737108; doi:10.1371/journal.pone.0071476)

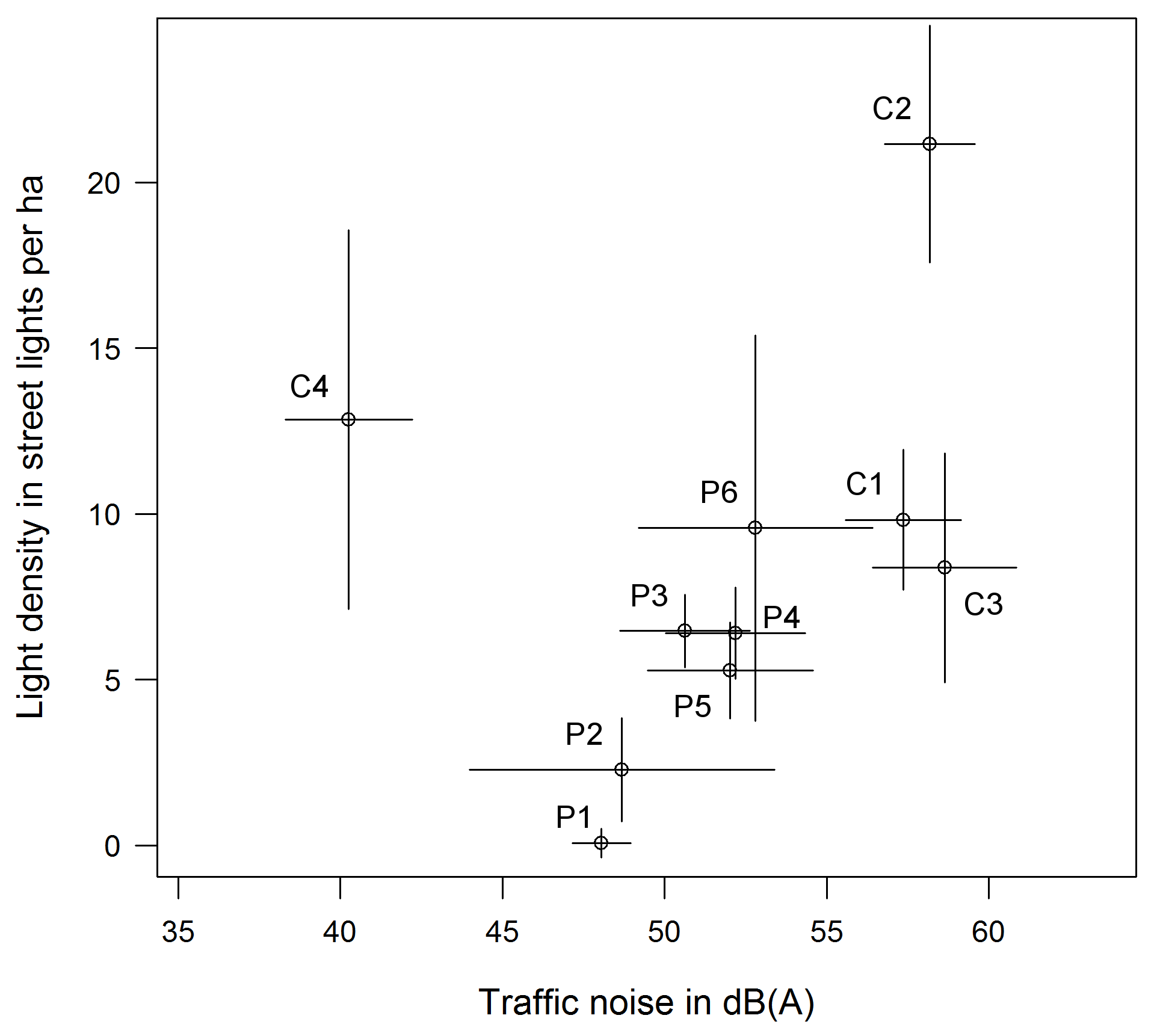

Supplement: Figure S1 — Study sites differ in traffic noise and artificial night light. The amount of traffic noise and artificial night light blackbirds experience at the song post are plotted as the mean and standard error for the corresponding study site. In the city centre, the sites show a high variability, whereas the bigger parks (P2– P5) are relatively homogenous. From the urban forest (P1) to the green spaces next to the ring road (C2) the noise and night light steadily increases, only the inner city centre deviates from the pattern by its low traffic noise. (TIFF) [file pone.0071476.s001.tiff]

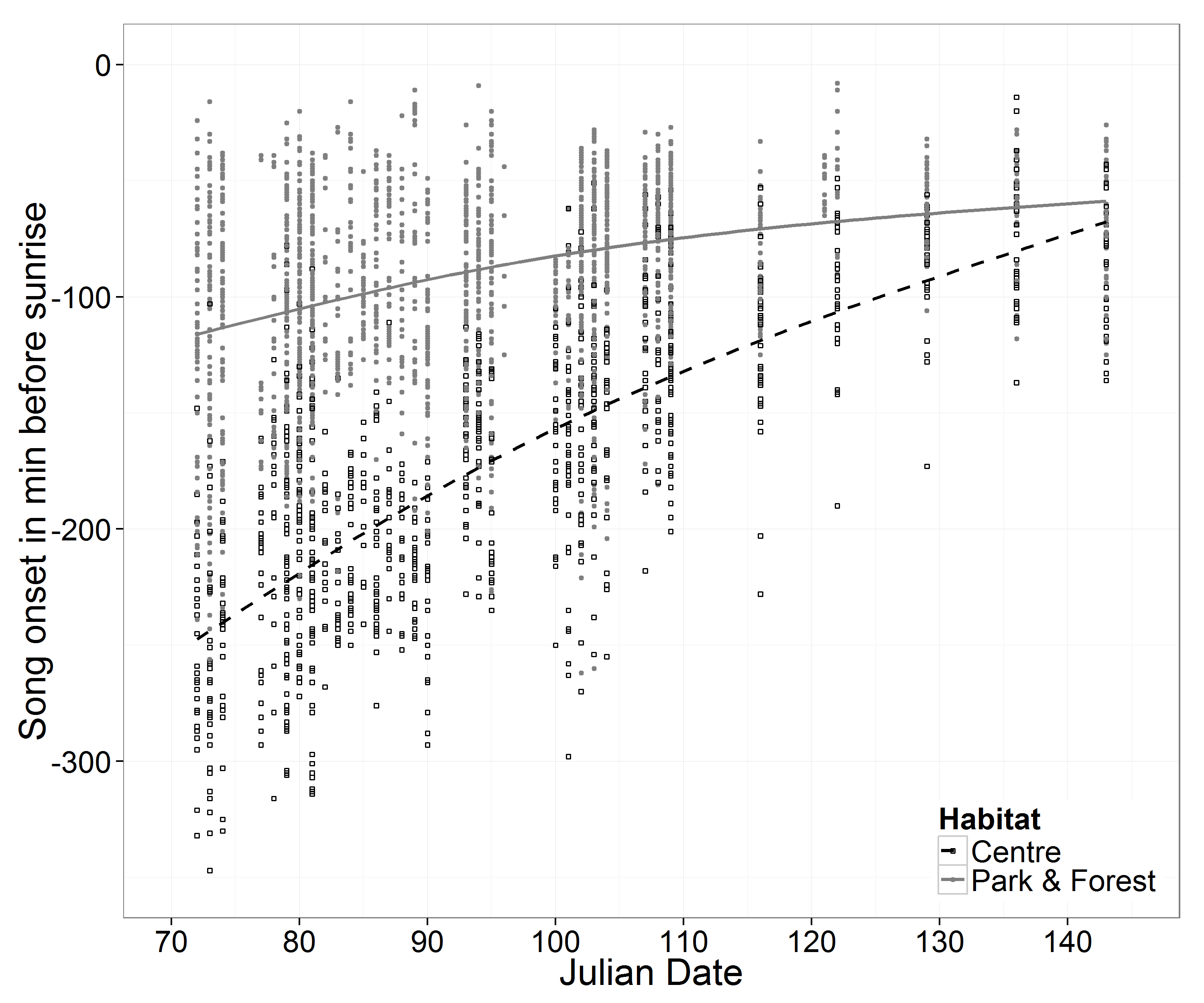

Supplement: Figure S2 — Delay of song onset of park and city centre blackbirds over the study period. A Julian Date of 90 indicates the 1st April 2011 and the 31st March 2012, respectively. (TIFF) [file pone.0071476.s002.tiff]

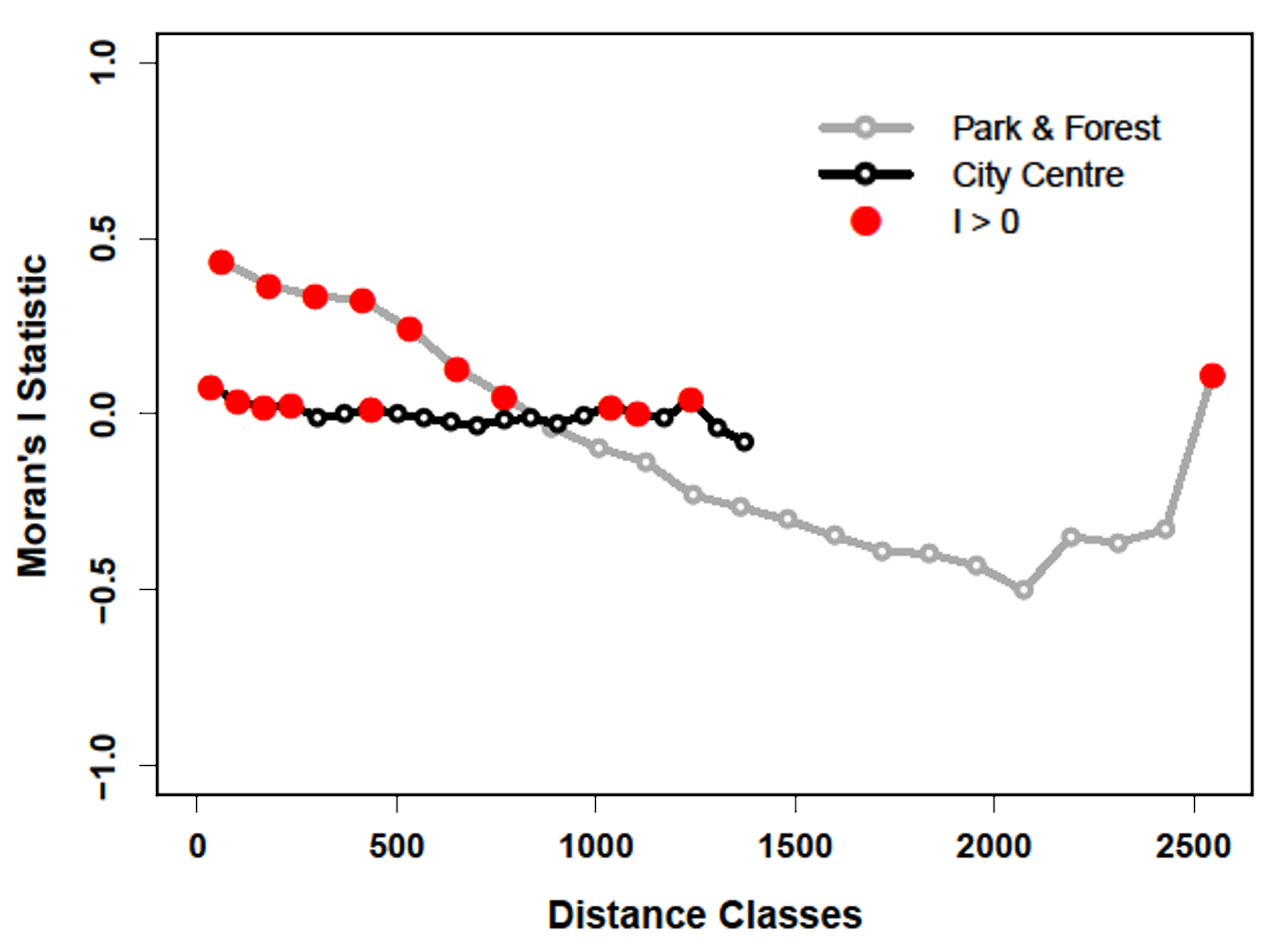

Supplement: Figure S3 — Moran’s I computed by distance class. Positive (negative) values indicate positive (negative) spatial autocorrelation. Values range from −1 (indicating perfect dispersion) to +1 (perfect correlation). A Moran’s I of zero indicates a random spatial pattern. Red points indicate spatial autocorrelation that is significant at the 5% level. (TIF) [file pone.0071476.s003.tif]
